# Real‐world evidence on disseminated intravascular coagulation from Japan

**DOI:** 10.1002/ams2.836

**Published:** 2023-04-09

**Authors:** Ryo Hisamune, Katsunori Mochizuki, Kazuma Yamakawa

**Affiliations:** ^1^ Department of Emergency and Critical Care Medicine Osaka Medical and Pharmaceutical University Osaka Japan

**Keywords:** Diagnosis procedure combination, disseminated intravascular coagulation, registry, retrospective study, sepsis

## Abstract

Many descriptive epidemiological and comparative studies using big data have been reported recently from outside Japan. Within Japan, diagnosis procedure combination (DPC) data and medical receipt data are being stored in electronic media, and real‐world evidence in various fields has started to be reported. We reviewed clinical studies on disseminated intravascular coagulation (DIC) using DPC data obtained from an insurance database with large numbers of cases and a related commercially available dataset including DPC and laboratory data. After DPC was introduced in 2003, 19 studies on DIC using Japanese national DPC data and two studies using the Medical Data Vision database were reported. Epidemiological findings in seven studies showed that the proportion of drugs administered for each underlying disease differed, with antithrombin and recombinant thrombomodulin (rTM) being used more frequently in clinical settings. In 14 comparative studies on anti‐DIC agents, antithrombin for severe pneumonia, postoperative intestinal perforation, and severe burn, and rTM for acute cholangitis were associated with improved survival rates. Large‐scale observational studies using big data can show results similar to those of randomized control trials if the quality of individual research is high. Real‐world data analysis will be increasingly necessary to complement the evidence gap unfilled by randomized control trials.

## Graphical abstract

Within Japan, diagnosis procedure combination (DPC) data and medical receipt data are being stored in electronic media, and real‐world evidence in various fields has started to be reported. We reviewed clinical studies on disseminated intravascular coagulation using DPC data obtained from an insurance database with large numbers of cases and a related commercially available dataset including DPC and laboratory data. Real‐world data analysis will be increasingly necessary to complement the evidence gap unfilled by randomized control trials.

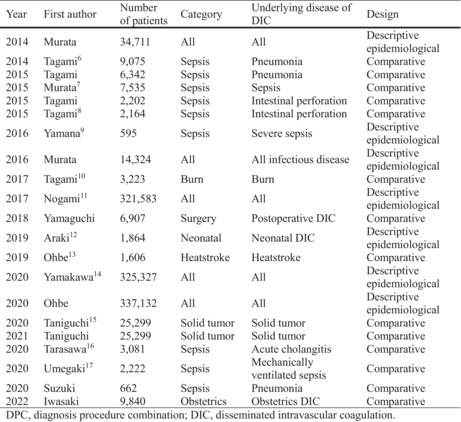

## INTRODUCTION

“Big data” is often described in terms of volume, velocity, and variety.^1^ The clinical field, which includes various information on diseases, drugs, and patient characteristics, is a treasure trove of big data. Recent remarkable advances in science and technology now allow huge data to be collected, stored, and managed. Big data in Japan can be broadly classified as patient registries, insurance databases, or electronic medical records data. Patient registries are databases that collect and register detailed information (laboratory data, vital signs, disease severity scores, etc.) on patients with specific disease from multiple centers, such as the Japan Trauma Data Bank led by the Japanese Association for the Surgery of Trauma. In contrast, insurance databases accumulate public medical insurance data such as receipt data and diagnosis procedure combination (DPC) data. Although fewer cases are generally collected from electronic medical records than from insurance databases, they can provide detailed laboratory and other clinical data.

Diagnosis procedure combination was introduced to 82 hospitals in 2003, and that number has gradually increased to 1,764 hospitals (approximately 480,000 beds) as of 2022.^2^ This covers approximately 92% of all acute‐care beds, and thus, DPC data provides the largest collection of clinical data in Japan. In this narrative review, we focus on clinical research on disseminated intravascular coagulation (DIC) using real‐world big data in Japan, which include the national DPC data and a commercially available dataset released from several companies. Disseminated intravascular coagulation is a critical status causing extremely systemic persistent activation of coagulation due to the underlying disease. In Japan, epidemiologic studies have been undertaken by a national ministry and the Japanese Society on Thrombosis and Hemostasis. Also, the use of anticoagulation therapy for DIC has been incorporated into clinical practice for the past few decades. As the number of DIC studies using the DPC database is recently increasing, we want to inform the world about the real‐world situation of DIC in Japan by summarizing the reports published to date, and discuss the current status and future issues in database research using big data.

## DISSEMINATED INTRAVASCULAR COAGULATION RESEARCH USING REAL‐WORLD DATA

In 2001, the International Society of Thrombosis and Hemostasis defined DIC as “a systemic intravascular coagulopathic active state caused by a variety of factors”.^3^ Studies on DIC patients with many underlying diseases have reported that patients with DIC have a mortality rate approximately twice that of patients without DIC.^4^ This has led to a better understanding of DIC pathophysiology and the need for a treatment strategy, which has resulted in well‐known treatment concepts that have reduced the mortality rate of DIC over the past 20 years.^5^ However, it is difficult to evaluate the effectiveness of DIC treatment in a simple randomized clinical trial (RCT) because treatment effect for DIC varies depending on patient pathophysiology and the underlying disease. Although several RCTs evaluating treatment effect in DIC patients have been carried out, there are currently no anticoagulants that have consistently shown efficacy.

Since the introduction of DPC in 2003, much clinical patient data have been accumulated to date. Nineteen studies on DIC using the Japanese national DPC data and two studies using the Medical Data Vision (MDV) database have been reported (Table 1, ^6^, ^7^, ^8^, ^9^, ^10^, ^11^, ^12^, ^13^, ^14^, ^15^, ^16^, ^17^ and File S1). These studies can be classified as either descriptive epidemiological studies or comparative studies on treatment and can be divided according to underlying diseases. However, a limitation of this study is that there is no formal search formula because it is a narrative review. We next introduce these studies according to study concept.

**Table 1:** Summary of diagnosis procedure combination studies on disseminated intravascular coagulation (DIC)

Year	First author	Number of patients	Category	Underlying disease of DIC	Design
2014	Murata	34,711	All	All	Descriptive epidemiological
2014	Tagami^6^	9,075	Sepsis	Pneumonia	Comparative
2015	Tagami	6,342	Sepsis	Pneumonia	Comparative
2015	Murata^7^	7,535	Sepsis	Sepsis	Comparative
2015	Tagami	2,202	Sepsis	Intestinal perforation	Comparative
2015	Tagami^8^	2,164	Sepsis	Intestinal perforation	Comparative
2016	Yamana^9^	595	Sepsis	Severe sepsis	Descriptive epidemiological
2016	Murata	14,324	All	All infectious disease	Descriptive epidemiological
2017	Tagami^10^	3,223	Burn	Burn	Comparative
2017	Nogami^11^	321,583	All	All	Descriptive epidemiological
2018	Yamaguchi	6,907	Surgery	Postoperative DIC	Comparative
2019	Araki^12^	1,864	Neonatal	Neonatal DIC	Descriptive epidemiological
2019	Ohbe^13^	1,606	Heatstroke	Heatstroke	Comparative
2020	Yamakawa^14^	325,327	All	All	Descriptive epidemiological
2020	Ohbe	337,132	All	All	Descriptive epidemiological
2020	Taniguchi^15^	25,299	Solid tumor	Solid tumor	Comparative
2021	Taniguchi	25,299	Solid tumor	Solid tumor	Comparative
2020	Tarasawa^16^	3,081	Sepsis	Acute cholangitis	Comparative
2020	Umegaki^17^	2,222	Sepsis	Mechanically ventilated sepsis	Comparative
2020	Suzuki	662	Sepsis	Pneumonia	Comparative
2022	Iwasaki	9,840	Obstetrics	Obstetrics DIC	Comparative

## DESCRIPTIVE EPIDEMIOLOGY STUDIES ON DIC

Six descriptive epidemiology studies used real‐world data obtained from Japanese national DPC data,^9^, ^12^, ^14^ and one study used the MDV database.^11^ Five studies focusing on overall DIC, one on sepsis‐induced DIC, and one on DIC in neonates are shown in Table 2. Studies using national DPC data included more than 10,000 patients and provided a powerful birds‐eye view of the epidemiology of DIC in Japan.

**Table 2:** Summary of descriptive epidemiology studies on disseminated intravascular coagulation (DIC)

Year	First author	Category	Number of patients	Research period (years)	Main findings
2014	Murata	All	34,711	3	Mortality rate decreased from year to year, with a significant decrease in sepsis
2016	Yamana^9^	Sepsis	595	1	DIC diagnosis based on DPC data has low sensitivity but high specificity
2016	Murata	Sepsis	14,324	3	Successively increasing proportion of rTM formulations administered
2017	Nogami^11^	All	321,583	4	Type 2 diabetes is a high risk for DIC
2019	Araki^12^	Neonatal	1,864	2	Incidence is 2.4% of hospitalized patients; risk factor is low birthweight
2020	Yamakawa^14^	All	325,327	8	Mortality rates have improved, and the percentage of rTM drugs administered has increased in each disease group
2020	Ohbe	All	460,448	8	Organ damage is more common in macrovascular/sepsis, and bleeding events are more common in macrovascular/obstetrics

DPC, diagnosis procedure combination; rTM, recombinant thrombomodulin.

A major limitation of the national DPC data is the lack of laboratory data, which prevents researchers from using commonly used DIC diagnostic criteria. In 2016, Yamana *et al*.^9^ published a study evaluating the validity of DIC diagnosis based on International Statistical Classfication of Disease and Related Health Problems (ICD‐10) codes compared to that based on the Japanese Association for Acute Medicine DIC scores using laboratory data. The sensitivity and specificity of the ICD‐10 codes for the diagnosis of DIC were 55% and 67%, respectively. From this first detection of the high specificity of DIC diagnosis by ICD‐10 coding, we began to undertake further DIC research using DIC cases diagnosed in the database.

Yamakawa *et al*.^14^ reported the epidemiology of 325,325 DIC patients with all underlying diseases over an 8‐year period. The overall mortality rate of DIC patients decreased successively from 41.8% to 36.1%, and that for each underlying disease showed a similar downward trend. To examine the trend in drug use for DIC treatment, several reports addressed the therapeutic effect on DIC of recombinant thrombomodulin (rTM),^18^, ^19^ whose use has increased dramatically since 2012. The results revealed the real‐world clinical situation, that rTM and antithrombin (AT) are currently the two most‐used drugs for DIC treatment in Japan. The strength of using the DPC database is that it can clarify the actual trend of big patient data.

One advantage of using the DPC is its strength in analyzing rare diseases. Araki *et al*.^12^ reported the first epidemiological study of 1,864 neonates with DIC in Japan. The incidence of DIC was 2.4%, and the mortality rate at DIC diagnosis was 14.1%. Risk factors for developing DIC were birth before 28 weeks of gestation and very low birthweight. Disseminated intravascular coagulation was a significant contributor to neonatal mortality and length of hospital stay.

Nogami *et al*.^11^ report on the association between DIC and type 2 diabetes using MDV data. Patients with type 2 diabetes diagnosed by laboratory test had a higher incidence of DIC (1.8% vs. 1.1%) and higher mortality rate (34.2% vs. 28.0%) compared to nondiabetic patients. This trend was similar for both DIC patients detected using receipt data and that based on laboratory data. The MDV data includes medications, date of prescription, medical treatments, and laboratory test results, so by using it, we can analyze the relation of data obtained from patients to DIC parameters, including outcome that are not available in the national DPC database.

In summary, descriptive epidemiology studies for DIC using real‐world big data mainly reported trends in overall mortality and anticoagulant use, and characteristics according to underlying diseases. The epidemiology of neonatal DIC, a rare disease whose investigation was previously difficult, was also examined. The DPC data allowed us to obtain an overview of DIC in actual clinical practice.

## COMPARATIVE STUDIES FOR ANTICOAGULANT THERAPIES

Many previous high‐impact articles on DIC treatments using national DPC data and the MDV are available as shown in Table 3.^6^, ^7^, ^8^, ^10^, ^12^, ^15^, ^16^, ^17^ Most evaluated were the association of the administration of AT or rTM, and that of their administration, with short‐term mortality.

**Table 3:** Summary of comparative studies on anti‐disseminated intravascular coagulation (DIC) therapeutic drugs

Year	First author	Underlying disease of DIC	Number of patients	Arm	Matching patient number	Primary outcome		
2014	Tagami^6^	Severe pneumonia	9,075	AT(+)	2,194	28‐day mortality	41.1%	*
				AT(−)	2,194		45.1%	*
2015	Tagami	Severe pneumonia	6,342	rTM(+)	1,140	28‐day mortality	37.6%	
				rTM(−)	1,140		37.0%	
2015	Murata^7^	Septic DIC	7,535	AT(+)	3,601	28‐day mortality	28.1%	
				rTM(+)	3,934		26.8%	
2015	Tagami	Sepsis after intestinal perforation	2,202	rTM(+)	621	28‐day mortality	26.1%	
				rTM(−)	621		24.8%	
2015	Tagami^8^	Sepsis after intestinal perforation	2,164	AT(+)	518	28‐day mortality	19.9%	*
				AT(−)	518		27.6%	*
2017	Tagami^10^	Severe burn	3,223	AT(+)	103	28‐day mortality	33.0%	*
				AT(−)	103		47.6%	*
2018	Yamaguchi	Post surgery	6,907	rTM	2,117	Bleeding complication	18.8%	*
				rTM(−)	2,117		24.8%	*
2019	Ohbe^13^	Heatstroke	1,606	rTM(+)	556	In‐hospital mortality	Risk difference −5.5%	*
				AT(+)	556		Risk difference −4.2%	
				AT, rTM(−)	494		Risk difference −6.5%	*
2020	Taniguchi^15^	Solid tumor (stage IV)	25,299	AT(+)	919	28‐day mortality	30.3%	
				AT(−)	3,676		28.9%	
2021	Taniguchi	Solid tumor (stage IV)	25,299	rTM(+)	1,979	28‐day mortality	34.3%	
				rTM(−)	7,916		37.4%	
2020	Tarasawa^16^	Acute cholangitis	3,081	rTM(+)	910	28‐day mortality	9.5%	*
				rTM(−)	910		12.9%	*
2020	Umegaki^17^	Mechanically ventilated sepsis	2,222	AT	1,017	28‐day mortality	53.2%	
				AT + rTM	1,205		54.8%	
2020	Suzuki	Severe pneumonia	662	rTM	189	In‐hospital mortality	45.5%	
				rTM + AT	189		40.2%	
2022	Iwasaki	Obstetrics DIC	9,840	AT(+)	3,290	28‐day mortality	0.3%	
				AT(−)	3,290		0.4%	

AT, antithrombin; rTM, recombinant human soluble thrombomodulin.

* *P* < 0.05 (significant difference).

The use of AT was evaluated on its beneficial association with mortality in several underlying diseases, such as severe pneumonia (41.4% vs. 45.1%),^6^ postoperative intestinal perforation (19.9% vs. 27.6%),^8^ and severe burn injury (33.0% vs. 47.6%).^10^ No significant reduction in mortality was observed with the use of AT for DIC caused by solid cancer, but a trend toward lower bleeding rates was observed.^15^

Anti‐DIC agent rTM was also evaluated for its treatment effect on DIC caused by several underlying diseases. Recombinant thrombomodulin use was significantly associated with improved mortality compared to its non‐use in DIC patients with acute cholangitis (9.5% vs. 12.9%)^13^ and heat shock (risk difference −5.5%; 95% confidence interval, −9.5% to −1.6%).^16^ Although not a propensity score‐matched analysis, one study for overall infections resulting in DIC found significant improvement in in‐hospital mortality (38.6% vs. 45.3%) and lower health‐care costs (€16,343 vs. €19,940) in the rTM group versus the AT group.^7^ In DIC cases with solid cancer, the rTM group did not differ significantly in the primary or secondary end‐points, similar to AT treatment.

Monotherapy and combination therapy were studied for AT and rTM. For mechanically ventilated sepsis‐induced DIC, AT monotherapy was compared with concomitant therapy of AT and rTM.^17^ There was no significant difference in the primary end‐point of 28‐day mortality or the secondary end‐point of hospital length of stay.

## DIFFERENCE IN STRENGTH BETWEEN REAL‐WORLD EVIDENCE AND RCTs

The universal gold standard in the evidence hierarchy of clinical research is the RCT.^20^ It is the best method to exclude various confounding factors by random assignment to an intervention and control group. Patient backgrounds between groups should be similar, intervention methods are homogenous, and arbitrariness in outcome assessment is eliminated. This provides a design with high internal validity that comes as close as possible to showing a true effect. However, ethical and cost issues, patient selection, and posttrial issues make it impractical to seek answers to clinical questions through conventional RCT design alone. Furthermore, because RCTs are often carried out under an experimental setting rather than in routine clinical practice, the target population and number of subjects are limited. Theoretical comparisons are made in an environment far from real‐world clinical settings. Especially, RCTs in critical care fields usually contain several limitations, as summarized by Granholm *et al*.^20^ Mortality is commonly the primary outcome in RCTs, and although important for patients, it conveys limited information; that is, if the intervention of interest has a clinical effect but does not sufficiently relate to death, it is difficult to detect statistical significance.

The difference between RCTs and real‐world clinical studies using DPC big data is summarized in Table 4. Studies that use currently available real‐world big data are carried out under low financial burden, and long‐term observation is possible.^21^ Large‐scale observational studies using real‐world big data can complement the evidence gap left unfilled by RCTs.^22^, ^23^ Many studies undertaken outside of Japan that use big data have been reported even in critical care fields.^24^ Currently, research using big data is still in its infancy in Japan, but understanding and knowledge of using big data will need to be shared in the future with clinicians.

**Table 4:** Features and differences between randomized clinical trials (RCTs), registry data, and diagnosis procedure combination (DPC) database

Setting	RCT	Registry data	DPC database
	Experimental environment	Real‐world	Real‐world
Evaluating content	Efficacy	Effectiveness	Effectiveness
Subject scale	Limited	Limited	Diverse
Target population	Small	Medium	Large (whole nation)
Confounding effect	Less susceptible	Susceptible	Susceptible
Internal validity	Superior	Inferior	Inferior
External validity	Inferior	Superior	Superior
Cost	Expensive	Inexpensive	Inexpensive
Observational period	Short	Long	Short
Practicable	Low	High	High
Data related			
Patient consent	Letter agreement	Unnecessary for secondary use	Unnecessary for secondary use
Database structure	Settled	Different from database	Settled
Database holder	Sponsor for RCTs	Academic conference etc.	Ministry of Health, Labour, and Welfare
Data cleaning	Already performed	Not performed	Not performed
Strong point	Verifying causality	Specific disease data	Abundant patient data
Weak point	Limited data	Sometimes lack of data	Mainly inpatient data

## LIMITATIONS OF DIC RESEARCH USING DPC DATASET

Due to the specific nature of DIC studies, there are several limitations of using DPC data: no laboratory data, limited background data, no data on treatment trends, and no data on prescription drugs or duration of drug use. It is more ambiguous to diagnose DIC by ICD‐10 code than by established criteria based on patient clinical findings and laboratory data. Information to assess details of organ failure and trends of laboratory data are also unavailable from the national DPC database. Commercially available data such as the MDV dataset are made by receipt from health insurance unions and health checkup data; however, data from all hospitals in Japan is lacking, making these data less representative. The DPC data provide limited patient background data, making it difficult to evaluate the effectiveness of treatments sufficiently and impossible to adjust patient background without unknown bias as can be done in RCTs.

To further develop and effectively utilize DPC data, the above limitations must be overcome. When assessing the validity of clinical practice for DIC in Japan, detailed clinical data must be collected that includes not only current patient and laboratory data available from the DPC but also detailed patient‐oriented data such as basic patient data, prescription data, in‐hospital data, and genetic data. As the importance of precision medicine increases, data analysis that leads to routine diagnosis, prediction of side‐effects, and treatment decisions will be undertaken with artificial intelligence and other sophisticated methods.^25^ We believe it necessary to construct more granular real‐world data in Japan that can overcome this limitation in the future.

## FUTURE PERSPECTIVES

Various RCTs have been carried out to evaluate the effects of anti‐DIC agents, but they remain unconfirmed. Recently, however, it has become clear that there may be a target population that requires anti‐DIC agents.^26^, ^27^ As undertaking large‐scale RCTs using anti‐DIC agents could become difficult in the future, searching for appropriate target populations for treatment from big data obtained from actual clinical settings might be a valid option. Attempts to link data from clinical practice to research, as is being done for other fields outside of Japan, will be required.

## DISCLOSURE

Approval of the research protocol: N/A.

Informed consent: N/A.

Registry and the registration no. of the study/trial: N/A.

Animal studies: N/A.

Conflict of interest: K.Y. reported receiving grants from Asahi Kasei Pharma and Nihon Pharmaceutical. The other authors declare no conflict of interest for this article.

## Supporting information


**File S1** Reference list of diagnosis procedure combination studies on disseminated intravascular coagulation not listed in manuscript.
