# Supplementary material for: Real‐world evidence on disseminated intravascular coagulation from Japan
Source: Acute Med Surg. 2023 Apr 9;10(1):e836. doi: 10.1002/ams2.836 (PMC10083461; doi:10.1002/ams2.836)
Supplement: Supplementary file 1 — File S1 Reference list of diagnosis procedure combination studies on disseminated intravascular coagulation not listed in manuscript. [file AMS2-10-e836-s001.docx]

**Supplementary file 1**

**Reference list of DPC studies on disseminated intravascular coagulation which was not listed in manuscript.**

- Murata A, Okamoto K, Mayumi T, Muramatsu K, Matsuda S. The recent time trend of outcomes of disseminated intravascular coagulation in Japan: an observational study based on a national administrative database. J. Thromb. Thrombolysis. 2014; 38: 364–71.
- Tagami T, Matsui H, Horiguchi H, Fushimi K, Yasunaga H. Recombinant human soluble thrombomodulin and mortality in severe pneumonia patients with sepsis-associated disseminated intravascular coagulation: an observational nationwide study. J. Thromb. Haemost. 2015; 13: 31–40.
- Tagami T, Matsui H, Fushimi K, Yasunaga H. Use of recombinant human soluble thrombomodulin in patients with sepsis-induced disseminated intravascular coagulation after intestinal perforation. Front. Med. (Lausanne). 2015; 2: 7.
- Murata A, Okamoto K, Mayumi T, Muramatsu K, Matsuda S. Recent change in treatment of disseminated intravascular coagulation in Japan: an epidemiological study based on a national administrative database. Clin. Appl. Thromb. Hemost. 2016; 22: 21–7.
- Yamaguchi T, Kitajima Y, Miyauchi Y*et al*. Assessment of bleeding in patients with disseminated intravascular coagulation after receiving surgery and recombinant human soluble thrombomodulin: a cohort study using a database. PLoS One. 2018; 3(10): e0205146.
- Ohbe H, Yamakawa K, Taniguchi K *et al*. Underlying disorders, clinical phenotypes, and treatment diversity among patients with disseminated intravascular coagulation. JMA J. 2020; 3: 321–9.
- Taniguchi K, Ohbe H, Yamakawa K, Matsui H, Fushimi K, Yasunaga H. Recombinant thrombomodulin in disseminated intravascular coagulation associated with stage IV solid tumors: a nationwide observational study in Japan. Thromb. Haemost. 2021; 121: 36–45.
- Suzuki J, Sasabuchi Y, Hatakeyama S *et al*. The effect of antithrombin added to recombinant human-soluble thrombomodulin for severe community-acquired pneumonia-associated disseminated intravascular coagulation: a retrospective cohort study using a nationwide inpatient database. J. Intensive Care. 2020; 8: 8.
- Iwasaki Y, Ohbe H, Shigemi D, Fushimi K, Yasunaga H. Effect of antithrombin III among patients with disseminated intravascular coagulation in obstetrics: a nationwide observational study in Japan. BJOG. 2022; 129: 805–11.
